# Developed diplopia due to a pituitary macroadenoma during pregnancy

**DOI:** 10.11604/pamj.2018.29.39.12706

**Published:** 2018-01-17

**Authors:** Houda Ennaifer, Manel Jemel, Hejer Kandar, Wafa Grira, Ines Kammoun, Leila Ben Salem

**Affiliations:** 1 Department of Endocrinology, National Institute of Nutrition, Tunis, Tunisia

**Keywords:** Diplopia, pituitary adenoma, pregnancy, ptosis

## Abstract

Physiologic pituitary enlargement is common during normal pregnancy. However, symptoms such as diplopia, blurred vision and headache resulting from physiologic pituitary enlargement are very rare during pregnancy. A 43-year-old woman complained of sudden headache and left eye ptosis at 36th weeks of gestation. An magnetic resonance imaging (MRI) demonstrated the pituitary enlargement and a macroadenoma without a compressing of the optic chiasm, but with an extension to the left cavernous sinus. 48 hours after the prescription of the bromocriptine, we had a spectacular evolution with disappearance of the headache and a total regression of the ptosis. We report a case of visual loss due to the physiologic pituitary enlargement or to the macroadenoma during pregnancy, which regressed after the prescription of bromocroptine.

## Introduction

The pituitary gland undergoes global hyperplasia during pregnancy because more estrogen stimulates hyperplasia of lactotrophes. Some studies have revealed that the size of pituitary gland increases an average of 120% during normal pregnancy [1]. The physiologic enlargement of pituitary gland during pregnancy usually does not expand to the extrasellar area to cause symptoms such as headache or blurred vision [1]. Prolactin secreting adenomas are the most commonly encountered pituitary tumors in women of child bearing age. However, undiagnosed pituitary tumor during pregnancy is not common, because prolactinoma is related with infertility or reproductive dysfunction [1]. We here report a rare case of developed left ptosis resulting from the enlargement of a pituitary adenoma during pregnancy that have not been diagnosed before pregnancy.

## Patient and observation

**Figure 1:**
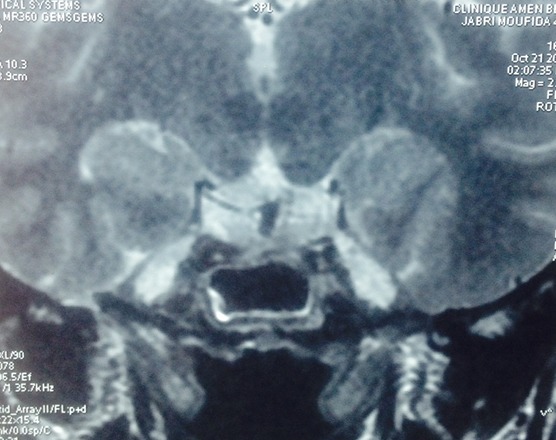
Magnetic resonance imaging (MRI) demonstrated a macroadenoma with the size of 1.35 ×2.9 ×1.2 cm, without a suprasellar extension or a compression of the optic chiasma. It encased the left cavernous sinus and internal carotid artery

A 43-year-old women visited our institution at 36th weeks of gestation with 2-Months history of headache and left ptosis. After a review by the obstetrician, she was admitted to our departement. We consulted neurosurgeon and ophthalmologist for a review. She had regular menstruation cycle before pregnancy and it is a spontaneous pregnancy. Therefore, she had not been examined any evaluations for pituitary gland or pituitary hormones before pregnancy. There were no specific past medical or operative histories. Ophtalmologic examination revealed a moderate left ptosis and the visual field was normal. Magnetic resonance imaging (MRI) demonstrated a macroadenoma with the size of 1.35 × 2.9 × 1.2cm, without a suprasellar extension or a compression of the optic chiasma. It encased the left cavernous sinus and internal carotid artery (Figure 1). Endocrine tests indicated that prolactin level was 6882 mUI/l (but our patient is pregnant). We practiced a 1 ug synacthene test and the cortisol levels were normal (T0 = 1100.92 nmol/l, T1 = 1256.39 nmol/l) excluding a cortitcotrope insufficiency. The patient had a thyreotrope insufficiency: FT4 = 3.35 pmol/l (7.8-14.4), TSH = 1.56 mUI/ml (0.34-5.6). We didn't explore the somatotropic axis. Obstetric ultrasound was normal and a cesarean was planned at 38th weeks of gestation. We initiated dopamine agonist therapy with 1.25 mg of bromocriptine once for 2 days, titrated to 1.25 mg twice daily. 48 hours after the prescription of the bromocriptine, we had a spectacular evolution with disappearance of the headache and a total regression of the ptosis. The patient gave birth to a healthy boy. They are both doing well. She is still on bromocriptine 1.25 mg twice daily and we didn't allow the breastfeeding. The prolactin and the MRI were planned at the first and the third month of the post partum.

## Discussion

The pituitary gland normally enlarges during pregnancy, predominantly because of the hyperplasia of prolactin producing lactotroph cells. Both estrogen and progesterone stimulate the synthesis of prolactin. Serum prolactin level is gradually increased with a parallel increase in the size and number of lactotroph cells during pregnancy [2]. The lactotroph hyperplasia starts in early pregnancy and then disappears spontaneously within 2 weeks to 6 months after delivery [3]. The size of the pituitary gland may expand an average of 120% during a normal pregnancy compared to prepregnancy [1]. It is difficult to differentiate the pathologic diagnoses of pituitary enlargement during pregnancy because MRI is not sufficiently sensitive to specific diseases. Moreover, a normally elevated prolactin level during pregnancy also hinders the diagnosis of pathologic pituitary gland [4]. The height of the pituitary gland is correlated to gestational age (0.08 mm per week) but it seldom increases to more than 10 mm during pregnancy [1]. Pituitary height higher than 9 to 10 mm during pregnancy may arouse suspicion of a pathological reason [5]. The pituitary gland of our patient had enlarged to 29 mm in height, which was larger than that of reported physiological enlargement of pituitary glands during pregnancy. Our patient's MRI demonstrated the asymmetric enlargement, which is not typical for physiologic enlargement of a pituitary gland. This finding may indicate that her pathologic pituitary mass may be adenoma. Interestingly nonfunctioning adenomas are very rare during pregnancy, since fertility is usually impaired. Prolactinoma is the most common cause of pituitary enlargement during pregnancy. Prolactinoma is responsive to bromocriptine treatment and resolves dramatically after delivery. Our patient's clinical presentation was compatible with prolactinoma, because her symptoms had improved with dopamine therapy.

## Conclusion

Actually, in our case, the definite diagnosis was remained uncertain. The evolution of prolactin as well as the MRI aspect of control will therefore be the determining elements to be able to determine the exact diagnosis in our patient. We speculated that the combined factors of physiologic enlargement and incidentally detected pituitary adenoma during pregnancy influenced on her sudden developed symptoms such as ptosis and headache during pregnancy. Fortunately, her symptoms resulting from mass effect resolved on dopamine agonist therapy. We here report a rare case of developed left ptosis resulting from the enlargement of a pituitary adenoma during pregnancy that have not been diagnosed before pregnancy.

## Competing interests

The authors declare no competing interests.
